# Relationship between Nontraumatic Shoulder Disorders and Neuropathic Pain: Retrospective Observational Analyses of Clinical Features and Background Factors

**DOI:** 10.1155/2023/6046746

**Published:** 2023-09-29

**Authors:** Tsuyoshi Sasaki, Hitoshi Shitara, Tsuyoshi Tajika, Tsuyoshi Ichinose, Noritaka Hamano, Masataka Kamiyama, Atsushi Yamamoto, Tsutomu Kobayashi, Kenji Takagishi, Hirotaka Chikuda

**Affiliations:** Department of Orthopaedic Surgery, Gunma University Graduate School of Medicine, Maebashi 371-8511, Japan

## Abstract

**Background:**

Accurate identification of neuropathic pain is necessary for appropriate treatment; however, the relationship between nontraumatic shoulder disorders and neuropathic pain remains unknown. Therefore, this retrospective observational study aimed to investigate the relationship, features, background factors, and prevalence of neuropathic pain among patients with nontraumatic shoulder disorders.

**Methods:**

We evaluated 198 patients who visited our outpatient clinic, which specializes in shoulder disorders, from April 2015 to March 2016. The patients' age, sex, affected side, diagnosis, and pain duration were recorded, and the results of physical examination, including passive range of motion, impingement sign, and muscular strength assessments, were analyzed. The presence of neuropathic pain was assessed using the painDETECT questionnaire. Participants were divided into two groups according to the presence of neuropathic pain. Pain intensity was assessed using a visual analog scale, and the patient's mental status was assessed using the short-form McGill Pain Questionnaire and Hospital Anxiety and Depression Scale. The scores were compared between the groups.

**Results:**

Neuropathic pain was observed in 7.6% of patients. The visual analog scale score for pain, short-form McGill Pain Questionnaire score, and Hospital Anxiety and Depression Scale score were significantly associated with the presence of neuropathic pain in the univariate analysis. Patient background factors and physical function were not associated with the presence of neuropathic pain. The prevalence of neuropathic pain in patients with frozen shoulder was 33.3%, which was significantly higher than that in patients with other shoulder disorders.

**Conclusion:**

The occurrence of neuropathic pain may aggravate pain in patients with nontraumatic shoulder disorders. Neuropathic pain was not a rare condition in patients with nontraumatic shoulder disorders, particularly in those with frozen shoulder. The coexistence of neuropathic pain cannot be determined from background factors or physical function. Accurate diagnosis of neuropathic pain is essential in patients with nontraumatic shoulder disorders.

## 1. Introduction

Neuropathic pain is defined as “pain caused by a lesion or disease of the somatosensory system” [1] and is associated with allodynia and hyperalgesia [2]. Generally, nociceptive pain and neuropathic pain have different clinical presentations and require different treatment approaches [3]. Often, neuropathic pain is unresponsive to traditional analgesics and is sometimes refractory to other pharmacotherapies [3–5]. Therefore, accurate identification of neuropathic pain is necessary for appropriate treatment. However, neuropathic pain is underestimated in many cases, resulting in inadequate treatment [6]. Chronic pain, including neuropathic pain, is underestimated worldwide, and it has been pointed out that correct pain assessment by specialists and scoring of pain intensity at each visit are needed [7]. However, a previous survey of anesthesiologists reported a low interest concerning the use of pain scales in the treatment of chronic pain [8], suggesting that chronic pain, including neuropathic pain, is difficult to treat. Adequate pain care should be regarded as a right and be effectively guaranteed to patients with chronic pain [9]. In this context, accurate evaluation and treatment of chronic pain and related neuropathic pain are desirable.

Phantom pain following limb amputation is a well-known example of neuropathic pain in patients with orthopedic disorders. Recently, neuropathic pain has been associated with nontraumatic orthopedic disorders, such as osteoarthritis of the knee or hip, lower back pain, and rheumatoid arthritis [10–12]. In shoulder disorders, neuropathic pain is known to be associated with rotator cuff tears. Although several studies have reported the relationship between neuropathic pain and pain intensity in patients with rotator cuff tears [13, 14], the relationship between neuropathic pain and other nontraumatic shoulder disorders, except for rotator cuff tears, was not clarified. Moreover, the nature of pain in patients with neuropathic pain has not been elucidated.

Therefore, this study aimed to determine the relationship between neuropathic pain and pain in various nontraumatic shoulder disorders and to identify characteristics and background factors in patients with neuropathic pain in shoulder disorders. We hypothesized that neuropathic pain is negatively associated with pain in patients with rotator cuff tears and in those with various nontraumatic shoulder disorders.

## 2. Materials and Methods

### 2.1. Ethics

The Institutional Ethical Review Board for Medical Research involving human subjects approved this cross-sectional observational study (approval number: 14-74). The research was performed in accordance with the tenets of the Declaration of Helsinki. All participants provided written informed consent after receiving an appropriate explanation of the study.

### 2.2. Participants

Patients who visited our outpatient clinic, which specializes in shoulder disorders, from April 2015 to March 2016 were retrospectively evaluated. The inclusion criterion was as follows: presence of nontraumatic shoulder disorders, such as degenerative rotator cuff tear or osteoarthritis. The exclusion criteria were as follows: history of previous trauma, previous surgery, or any other treatment to the affected shoulder; cervical spine disorders or neuromuscular disease; bilateral cases; and refusal to participate. The study population comprised 118 male and 80 female individuals (average age: 62.7 years; standard deviation (SD): 11.9 years; range: 26–85 years). The mean duration of pain was 8.3 months (SD: 15.4 months; range: 0.03–132.0 months). Based on the International Association for the Study of Pain definition of chronic pain (pain lasting >3 months), 80 patients had acute pain and 118 had chronic pain.

### 2.3. Evaluated Items

#### 2.3.1. Demographic Characteristics, Clinical Background, and Physical Examinations

The following patient characteristics were recorded: age, sex, affected side, and duration of pain. Physical examination of participants was conducted to evaluate the passive range of motion (ROM), muscular strength, and impingement signs. The upper extremity ROM was evaluated in four positions (ROM in flexion, abduction, external rotation, and internal rotation), with the patient in the sitting position. The internal rotation ROM was assessed based on the highest spinal segment that patients could reach with their thumb. The spinal segment was converted to a numerical value, as described previously [15]. Muscular strength was assessed during abduction and external rotation. In particular, muscular strength during abduction was assessed with the patient's arm at 90° of abduction and the palm down, whereas muscular strength during external rotation was assessed with the arm at the side, with 90° of elbow flexion, and a neutral position in pronation/supination. The peak isometric muscular strength was assessed using a digital myodynamometer (Micro FET, Hoggan Health Industries Inc., West Jordan, UT, USA) positioned at the distal end of the arm. The impingement sign was assessed in the Neer and Hawkins positions [16, 17]; it was considered positive when impingement sign positivity was observed in at least one of these positions.

### 2.4. Radiographic Evaluation

Plain radiographs of both shoulders were obtained in anterior-posterior, scapular-Y view, and axial directions. Magnetic resonance imaging (MRI) or ultrasound examination was performed when deemed necessary by the treating physician to evaluate soft tissue status. Computed tomography was performed when it was necessary to evaluate bone morphology.

### 2.5. Shoulder Disorder Diagnosis

Diagnosis was guided by a combination of physical examination and imaging findings. A rotator cuff tear was identified when a rotator cuff footprint avulsion was visible on MRI or ultrasound. Subacromial impingement syndrome was diagnosed when patients had a positive subacromial impingement sign [16, 17] without rotator cuff avulsion. A frozen shoulder was diagnosed when there was (1) history of pain and stiffness for >1 month, (2) passive ROM limitation <100° of flexion and <10° of external rotation and internal rotation (fifth lumbar vertebra or below), and (3) normal radiographic appearance [18]. Osteoarthritis was diagnosed if the patient had an osteoarthritic change with the Samilson–Prieto classification ≥1 on plain anterior-posterior radiography [19, 20]. Calcific tendinitis was diagnosed if the patient had calcification in the subacromial space on plain anterior-posterior radiography [21].

### 2.6. Neuropathic Pain Diagnosis

Neuropathic pain is usually diagnosed based on responses to a self-reported questionnaire regarding pain, such as painDETECT [22], which has been used worldwide for neuropathic pain diagnosis in various clinical settings [23]. In this study, the Japanese version of painDETECT was adopted for diagnosis of neuropathic pain. The validity and reliability of this version have been demonstrated previously [24], and it is widely used in Japan [25, 26]. The study patients completed the questionnaire during their first hospital visit. Using painDETECT, neuropathic pain is defined as a total score of ≥19 points [22]; this threshold was used to evaluate neuropathic pain prevalence in patients with shoulder disorders.

### 2.7. Pain Severity Assessment

Pain severity was assessed using a 100 mm visual analog scale (VAS) during the patients' first hospital visit. Patients were asked about their pain intensity over the preceding 3 days, including the day of the first visit. Pain at rest, pain on motion, and pain during the night were assessed separately. Using the questions in the painDETECT, pain at the first visit as well as the most severe pain and average pain experienced in the past 4 weeks before the first visit were also investigated using a numerical rating scale.

### 2.8. Patient's Mental Status Assessment

Patients' distress was assessed on the first visit using the Japanese version of the short-form McGill Pain Questionnaire (SF-MPQ) [27]. The SF-MPQ pain scale comprises 15 questions related to patients' distress due to pain and was originally written in English. The Japanese version of the SF-MPQ has been proven to be reliable, valid, and equivalent to the original questionnaire [28]. Patients rate each question on a 4-point intensity scale as follows: 0 = none, 1 = mild, 2 = moderate, and 3 = severe. The questions are divided into two components to assess the intensity of pain and the affected mental status separately. In this study, both the total score and the scores of each component were evaluated.

The patients' mental status was evaluated during the first hospital visit using the Hospital Anxiety and Depression Scale (HADS) [29], which has been widely used for diagnosing depression and anxiety [30]. In this study, both HADS-depression (HADS-D) and HADS-anxiety (HADS-A) were used [29, 31].

### 2.9. Neuropathic Pain Prevalence

The prevalence of neuropathic pain was studied for the entire study population and for patients with each shoulder disorder. First, this prevalence was evaluated in all participants, and then, it was computed and compared for each nontraumatic shoulder disorder.

### 2.10. Statistical Analysis

The participants were divided into two groups according to the presence or absence of neuropathic pain: groups with and without neuropathic pain. Patient demographics and all scale scores were compared between these two groups. Continuous variables were compared using the *t*-test or Mann–Whitney *U* test. Categorical variables were compared using the chi-squared test or Fisher's exact test.

As the outpatient data were retrospectively analyzed, no prior power analysis was performed for recruitment. Instead, effect sizes and confidence intervals for *t*-tests between the two groups were described to statistically examine the impact of neuropathic pain on pain in patients with nontraumatic shoulder disorders. Furthermore, to determine whether there were any differences in the prevalence of neuropathic pain among patients with five nontraumatic shoulder disorders, the prevalence for each disorder was compared using Fisher's exact test adjusted with Bonferroni's method.

All statistical analyses were conducted using SPSS version 26 (IBM Japan, Tokyo, Japan). Results are considered statistically significant at *P* values <0.05.

## 3. Results

**Figure 1:**
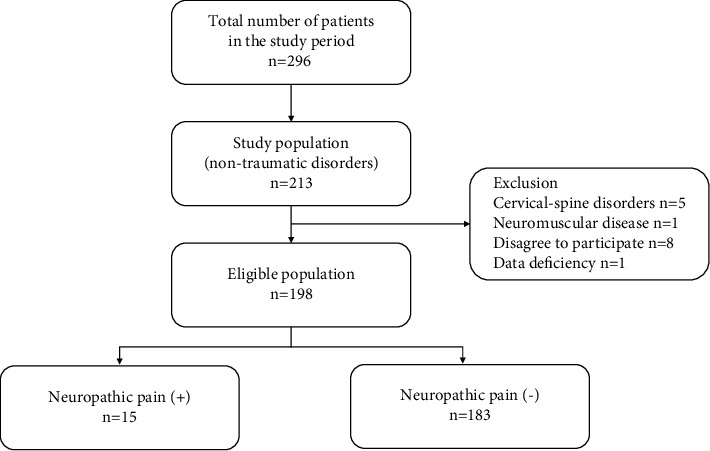
Flow diagram of patients included in the study based on the presence or absence of neuropathic pain. Overall, 93% of patients who attended our clinic during the study period with nontraumatic shoulder disorders were included in our study. Neuropathic pain was observed in 7.6% of the participants.

A total of 296 patients visited our outpatient clinic during the study period. Of these, 213 had nontraumatic shoulder disorders. Fifteen patients were excluded from the study, as they met the exclusion criteria, had missing data, or did not agree to participate. In total, 93.0% of patients who attended our clinic during the study period with nontraumatic shoulder disorders were included (Figure 1).

### 3.1. Demographics and Features of Patients with Neuropathic Pain

All patients completed all questionnaires regarding demographic characteristics and clinical background, as well as physical examination tests. Neuropathic pain was observed in 7.6% of subjects (*n* = 15). No differences in background factors were noted between patients with and without neuropathic pain (Table 1).  Table 2 presents the details of participants' diagnoses.

Pain at rest, pain on motion, and pain during the night were significantly more severe in patients with neuropathic pain. The effect sizes and 95% confidence intervals of the differences between the two groups in mean pain intensity at rest, on motion, and during the night are shown in Table 3. The most severe pain in the past 4 weeks before the first visit was not different between the two groups. In contrast, the average pain during the past 4 weeks was significantly more severe in patients with neuropathic pain. The analysis of the SF-MPQ results revealed that patients with neuropathic pain had higher total, sensation, and emotion scores (Table 1). There was a statistically significant difference in the total HADS scores between the two groups (Table 1). Patients with neuropathic pain had a higher HADS-D score than those without neuropathic pain; however, there were no statistically significant differences in the HADS-A scores between the two groups. Physical examinations did not reveal any significant differences between these two groups (Table 1).

### 3.2. Neuropathic Pain Prevalence in Different Shoulder Disorders

The prevalence of neuropathic pain was shown to be unevenly distributed according to Fisher's exact test. This prevalence was significantly higher in patients with frozen shoulder than in patients with other shoulder disorders after Bonferroni's adjustment (Table 4).

## 4. Discussion

In this study, we examined the relationship, features, background factors, and prevalence of neuropathic pain in patients with nontraumatic shoulder disorders. The most important findings were that 7.6% of patients with shoulder disorders were affected by neuropathic pain and that neuropathic pain was negatively associated with pain in patients with rotator cuff tears and in those with various nontraumatic shoulder disorders, as hypothesized. The VAS scores for pain at rest, on motion, and during the night and the SF-MPQ and HADS scores were significantly associated with neuropathic pain. There were no differences in background factors or in the results of physical examination between patients with and without neuropathic pain. The prevalence of neuropathic pain in patients with frozen shoulder was significantly higher than that in patients with other shoulder disorders.

### 4.1. Neuropathic Pain in Orthopedic Disorders

Phantom limb pain after amputation is a typical example of neuropathic pain in patients with orthopedic disorders, and the pathology may include both peripheral nerve injury caused by surgery and disorders of the central nervous system [32]. Conversely, some nontraumatic orthopedic disorders, which are not necessarily associated with direct nerve injury, such as osteoarthritis of the knee and hip, lower back pain, and rheumatoid arthritis, are known to be associated with neuropathic pain [10, 12, 33–35].

Karasugi et al. reported the characteristics of neuropathic pain using the painDETECT questionnaire in patients with rotator cuff tears and identified that the VAS score for pain in the previous 4 weeks was a factor in discriminating neuropathic pain [13]. Ko et al. investigated the characteristics of neuropathic pain in patients undergoing rotator cuff repair and reported that the VAS score for pain in the previous 4 weeks and tear size were factors in discriminating neuropathic pain [14]. However, we found no significant difference in the severity of pain in the previous 4 weeks between the groups with and without neuropathic pain among patients with rotator cuff tear in the present study. This difference may be attributed to the difference in population between the studies. In contrast, as these previous studies only evaluated the data of patients with rotator cuff tear, the relationship between neuropathic pain and shoulder disorders excluding rotator cuff tears has not been elucidated. Moreover, although they reported the intensity of pain in patients with neuropathic pain, the nature of the pain was not examined adequately. In the present study, neuropathic pain was diagnosed using painDETECT in both the overall population and in patients with each type of shoulder disorder, and the nature of pain was investigated in detail, which are the strengths of our study. The coexistence of neuropathic pain was negatively associated with pain severity in patients with general shoulder disorders, suggesting that neuropathic pain medications may be useful in the treatment of general shoulder disorders in some cases and that further research is warranted.

### 4.2. Neuropathic Pain Features in Shoulder Disorders

#### 4.2.1. Background Factors

Patients' background factors were not associated with the presence of neuropathic pain, which is consistent with the findings of previous reports that patients' background factors were not correlated with a neuropathic pain component in patients with orthopedic disorders [10, 13, 14, 33, 36]. Therefore, neuropathic pain may affect patients with orthopedic diseases regardless of background factors, indicating the importance for physicians to recognize the difficulty in predicting the coexistence of neuropathic pain based on patients' background factors alone.

#### 4.2.2. Pain Duration

Pain duration was not correlated with the presence of neuropathic pain. The temporal change in pain signaling is known to contribute to the development of chronic neuropathic pain [4, 6]. In general population surveys, pain duration was correlated with a neuropathic component [37, 38]. However, pain location in these surveys included multiple sites, ranging from the head to lower limbs. Therefore, the correlation between pain duration and neuropathic components in different disorders remains unclear. Further investigation is needed to clarify how pain duration affects neuropathic pain modulation in patients with shoulder disorders.

#### 4.2.3. Pain Intensity

In this study, patients with neuropathic pain reported more severe pain at rest, on motion, and during the night. This is consistent with the established clinical characteristics of neuropathic pain in patients with low back pain, carpal tunnel syndrome, and other chronic pain [10, 36, 39]. Furthermore, as the coexistence of neuropathic pain amplifies the intensity of pain during the night, neuropathic pain could be one of the reasons for pain during the night in patients with shoulder disorders. Further investigation is needed to clarify the correlation between neuropathic aberrations and pain during the night in patients with shoulder disorders.

#### 4.2.4. Mental Status

The SF-MPQ scores were higher in patients with neuropathic pain than in those without neuropathic pain. The SF-MPQ consists of two components that assess both the intensity of pain and an altered mental status. In this study, scores for both the components were higher in patients with neuropathic pain than in those without neuropathic pain. These results may suggest that the coexistence of neuropathic pain aggravates not only the intensity of pain but also the patient's mental health status. Furthermore, the total HADS score and HADS-D score were higher in patients with neuropathic pain than in those without neuropathic pain. Previous studies have described a correlation between neuropathic pain and a depressive state [40–42]; our results were consistent with the findings of these studies. Thus, in the neuropathic pain treatment, it is also important to consider the patient's mental status.

### 4.3. Neuropathic Pain Prevalence

Previous studies on orthopedic disorders have reported that the prevalence of neuropathic pain is approximately 12%–15% in patients with low back pain [10, 33] and 23% in patients with osteoarthritis of the knee or hip [11]. In this study, neuropathic pain was observed in 7.6% of the study population, with the highest prevalence observed in patients with frozen shoulder (33.3%), followed by patients with osteoarthritis (16.7%), calcific tendinitis (16.7%), rotator cuff tear (5.0%), and impingement syndrome (4.8%). These results indicate that, similar to that in patients with other orthopedic diseases, neuropathic pain is a common condition among patients with nontraumatic shoulder disorders, highlighting the importance of correctly diagnosing and adequately treating neuropathic pain in patients with shoulder disorders.

### 4.4. Differences in Neuropathic Pain between Rotator Cuff Tear and Frozen Shoulder

The symptoms of early stage rotator cuff tear and frozen shoulder are similar. Therefore, it is often difficult to diagnose these conditions based on physical findings alone without MRI results. In this study, when comparing the different shoulder disorders, the prevalence of neuropathic pain in patients with frozen shoulder was more than six times higher than that in patients with rotator cuff tear. Neuropathic aberrations detected by painDETECT can be used as supporting evidence to differentiate between rotator cuff tear and frozen shoulder.

### 4.5. Limitations

This study has some limitations. First, we only used a self-descriptive questionnaire to diagnose neuropathic pain. Future studies should objectively evaluate neuropathic pain, e.g., by confirming the presence of sensory abnormalities consistent with innervation. Second, patients' health behaviors, such as smoking and alcohol consumption, as well as the presence or absence of underlying diseases, such as hypertension and diabetes, were not investigated. Thus, the potential influence of these factors on neuropathic pain in patients with shoulder disorders was not clarified. Third, a multivariate analysis could not be performed owing to the small number of neuropathic pain-positive patients. Fourth, this study was performed at a single institution; therefore, larger multi-institutional studies are needed.

Nevertheless, this study revealed that neuropathic pain in patients with nontraumatic shoulder disorders is not a rare condition. This finding may help ensure better pain control in these patients.

## 5. Conclusions

We assessed the impact, features, and prevalence of neuropathic pain in 198 patients with nontraumatic shoulder disorders. Neuropathic pain was observed in 7.6% of participants. The occurrence of neuropathic pain may aggravate pain in patients with shoulder disorders. However, the coexistence of neuropathic pain could not be detected from the patients' background or physical examination findings alone. Neuropathic pain in patients with shoulder disorders was not a rare condition, particularly in patients with frozen shoulder. Clinicians should ensure that neuropathic pain is accurately diagnosed in patients with shoulder disorders.

## Figures and Tables

**Table 1:** Patient demographics and differences between patients with (+) and without (−) neuropathic pain.

	All patients	Nep (+)	Nep (−)	*P* value
Age (years)	62.7 ± 11.9	60.5 ± 13.9	62.9 ± 11.7	0.441
Sex (%)				
Male/female	59.6/40.4	53.3/46.7	60.1/39.9	0.598
Affected side (%)				
Dominant/nondominant	62.1/37.9	50.0/50.0	63.1/36.9	0.393
Pain duration (months)	8.3 ± 15.4	6.8 ± 8.4	8.5 ± 15.8	0.685
VAS score (mm)				
Rest	21.7 ± 23.1	44.5 ± 22.9	19.8 ± 22.1	**<0.001**
Motion	57.8 ± 27.2	73.1 ± 19.7	56.5 ± 27.4	**0.023**
Night	40.4 ± 30.5	59.1 ± 25.5	38.7 ± 30.4	**0.012**
painDETECT (points)	9.3 ± 7.1	24.6 ± 10.3	8.0 ± 5.1	**<0.001**
Pain at the first visit	4.8 ± 2.3	6.3 ± 2.2	4.6 ± 2.3	**0.012**
Most severe pain in the past 4 weeks	7.0 ± 2.4	7.6 ± 1.9	6.9 ± 2.4	0.294
Average pain during the past 4 weeks	5.0 ± 2.2	6.5 ± 2.3	4.9 ± 2.1	**0.011**
SF-MPQ score (points)				
Total	9.3 ± 8.4	16.9 ± 11.4	8.6 ± 7.8	**0.003**
Sensation	7.5 ± 6.3	13.2 ± 7.9	7.0 ± 5.9	**0.001**
Emotion	1.8 ± 2.5	3.7 ± 3.7	1.6 ± 2.3	**0.014**
HADS score (points)				
Total	7.8 ± 7.7	11.7 ± 7.6	7.5 ± 7.6	**0.024**
HADS-D	4.2 ± 4.2	6.5 ± 4.5	4.0 ± 4.2	**0.025**
HADS-A	3.6 ± 4.0	5.2 ± 3.9	3.5 ± 4.0	0.116
ROM (°)				
Flexion	131.6 ± 29.8	115.4 ± 41.4	132.9 ± 28.6	0.159
Abduction	123.2 ± 35.9	115.8 ± 32.8	123.7 ± 36.1	0.463
External rotation	39.2 ± 20.9	33.8 ± 25.8	39.6 ± 20.5	0.337
Internal rotation	12.1 ± 4.6	13.8 ± 5.5	11.9 ± 4.5	0.19
Muscle strength (kg)				
Abduction	3.5 ± 2.5	3.7 ± 2.8	3.5 ± 2.4	0.801
External rotation	4.4 ± 2.6	4.3 ± 2.7	4.4 ± 2.6	0.914

Values are presented as means ± standard deviations; *P* values represent the results of statistical comparisons between the groups with and without neuropathic pain. The VAS scores for pain, SF-MPQ score, and HADS score were significantly associated with the presence of neuropathic pain. Patient background factors and physical function were not associated with the presence of neuropathic pain. Nep, neuropathic pain; VAS, visual analog scale; SF-MPQ, short-form McGill pain questionnaire; HADS, Hospital Anxiety and Depression Scale; HADS-D, HADS-depression; HADS-A, HADS-anxiety; ROM, range of motion. The *P* value is bolded to emphasize that the difference is statistically significant (*P* value <0.05).

**Table 2:** Categorization of shoulder disorders.

Diagnosis	*N*
Rotator cuff tear	139
Impingement syndrome	21
Frozen shoulder	9
Osteoarthritis	6
Calcific tendinitis	6
Others	17

Nontraumatic shoulder disorders included rotator cuff tear, subacromial impingement syndrome, frozen shoulder, osteoarthritis, calcific tendinitis, and other conditions.

**Table 3:** The effect sizes and 95% confidence intervals of the differences in mean pain intensity at rest, on motion, and during the night between the two groups.

	*d*	95% CI (lower limit and upper limit)
*VAS score (mm)*		
Rest	1.1	−36.5, −13.0
Motion	0.7	−30.9, −2.3
Night	0.7	−36.3, −4.4

The 95% confidence interval for the difference in mean pain intensity between the two groups does not include 0, indicating that pain intensity was higher in patients with neuropathic pain than in patients without neuropathic pain. VAS, visual analog scale; *d*, Cohen's *d* (effect size); CI, confidence interval.

**Table 4:** Prevalence of neuropathic pain in patients with shoulder disorders.

Diagnosis	Number of cases (nep-positive/all)	Rate (%)
Frozen shoulder	3/9	33.3^*∗*^
Osteoarthritis	1/6	16.7
Calcific tendinitis	1/6	16.7
Rotator cuff tear	7/139	5.0
Impingement syndrome	1/21	4.8

^*∗*^Significant according to Fisher's exact test with Bonferroni's adjustment. The prevalence of neuropathic pain was significantly higher in patients with frozen shoulder than in patients with other shoulder disorders. Nep, neuropathic pain.

## Data Availability

The datasets generated and/or analyzed in the course of the current study are not publicly available following guidance from the Ethics Committee and are available from the corresponding author upon reasonable request.

## References

[1] Colloca L., Ludman T., Bouhassira D. (2017). Neuropathic pain. *Nature Reviews Disease Primers*.

[2] Jensen T. S., Finnerup N. B. (2014). Allodynia and hyperalgesia in neuropathic pain: clinical manifestations and mechanisms. *The Lancet Neurology*.

[3] Baron R., Gierthmühlen J. (2016). Neuropathic pain. *Seminars in Neurology*.

[4] Finnerup N. B., Attal N., Haroutounian S. (2015). Pharmacotherapy for neuropathic pain in adults: a systematic review and meta-analysis. *The Lancet Neurology*.

[5] Pickering G., Morel V. (2018). Memantine for the treatment of general neuropathic pain: a narrative review. *Fundamental & Clinical Pharmacology*.

[6] Harden N., Cohen M. (2003). Unmet needs in the management of neuropathic pain. *Journal of Pain and Symptom Management*.

[7] Szewczyk A. K., Jamroz-Wiśniewska A., Haratym N., Rejdak K. (2022). Neuropathic pain and chronic pain as an underestimated interdisciplinary problem. *International Journal of Occupational Medicine & Environmental Health*.

[8] Vittori A., Cascella M., Petrucci E. (2023). Strategies to build and maintain competence in pain management: insights from a SIAARTI survey on educational needs among Italian anesthesiologists. *Pain Practice*.

[9] Natoli S., Vittori A., Cascella M. (2022). Raising awareness on the clinical and social relevance of adequate chronic pain care. *International Journal of Environmental Research and Public Health*.

[10] Hiyama A., Watanabe M., Katoh H., Sato M., Sakai D., Mochida J. (2015). Evaluation of quality of life and neuropathic pain in patients with low back pain using the Japanese Orthopedic Association Back Pain Evaluation Questionnaire. *European Spine Journal*.

[11] French H. P., Smart K. M., Doyle F. (2017). Prevalence of neuropathic pain in knee or hip osteoarthritis: a systematic review and meta-analysis. *Seminars in Arthritis and Rheumatism*.

[12] Salaffi F., Di Carlo M., Carotti M., Sarzi-Puttini P. (2019). The effect of neuropathic pain symptoms on remission in patients with early rheumatoid arthritis. *Current Rheumatology Reviews*.

[13] Karasugi T., Ide J., Kitamura T., Okamoto N., Tokunaga T., Mizuta H. (2016). Neuropathic pain in patients with rotator cuff tears. *BMC Musculoskeletal Disorders*.

[14] Ko S., Choi C., Kim S., Chae S., Choi W., Kwon J. (2018). Prevalence and risk factors of neuropathic pain in patients with a rotator cuff tear. *Pain Physician*.

[15] Hamano N., Yamamoto A., Shitara H. (2017). Does successful rotator cuff repair improve muscle atrophy and fatty infiltration of the rotator cuff? A retrospective magnetic resonance imaging study performed shortly after surgery as a reference. *Journal of Shoulder and Elbow Surgery*.

[16] Hawkins R. J., Kennedy J. C. (1980). Impingement syndrome in athletes. *The American Journal of Sports Medicine*.

[17] Neer C. S. (1983). Impingement lesions. *Clinical Orthopaedics and Related Research*.

[18] Itoi E., Arce G., Bain G. I. (2016). Shoulder stiffness: current concepts and concerns. *Arthroscopy: The Journal of Arthroscopic & Related Surgery*.

[19] Kobayashi T., Takagishi K., Shitara H. (2014). Prevalence of and risk factors for shoulder osteoarthritis in Japanese middle-aged and elderly populations. *Journal of Shoulder and Elbow Surgery*.

[20] Samilson R. L., Prieto V. (1983). Dislocation arthropathy of the shoulder. *The Journal of Bone and Joint Surgery*.

[21] Hurt G., Baker C. L. (2003). Calcific tendinitis of the shoulder. *Orthopedic Clinics of North America*.

[22] Freynhagen R., Baron R., Gockel U., Tölle T. R. (2006). painDETECT: a new screening questionnaire to identify neuropathic components in patients with back pain. *Current Medical Research and Opinion*.

[23] Freynhagen R., Tölle T. R., Gockel U., Baron R. (2016). The painDETECT project-far more than a screening tool on neuropathic pain. *Current Medical Research and Opinion*.

[24] Matsubayashi Y., Takeshita K., Sumitani M. (2013). Validity and reliability of the Japanese version of the painDETECT questionnaire: a multicenter observational study. *PLoS One*.

[25] Inoue S., Taguchi T., Yamashita T., Nakamura M., Ushida T. (2017). The prevalence and impact of chronic neuropathic pain on daily and social life: a nationwide study in a Japanese population. *European Journal of Pain*.

[26] Takahashi N., Shirado O., Kobayashi K., Mashiko R., Konno S. (2016). Classifying patients with lumbar spinal stenosis using painDETECT: a cross-sectional study. *BMC Family Practice*.

[27] Melzack R. (1987). The short-form McGill pain questionnaire. *Pain*.

[28] Snaith R. P. (2003). The hospital anxiety and depression scale. *Health and Quality of Life Outcomes*.

[29] Bjelland I., Dahl A. A., Haug T. T., Neckelmann D. (2002). The validity of the hospital anxiety and depression scale. *Journal of Psychosomatic Research*.

[30] Djukanovic I., Carlsson J., Årestedt K. (2017). Is the Hospital Anxiety and Depression Scale (HADS) a valid measure in a general population 65-80 years old? A psychometric evaluation study. *Health and Quality of Life Outcomes*.

[31] Hsu E., Cohen S. P. (2013). Postamputation pain: epidemiology, mechanisms, and treatment. *Journal of Pain Research*.

[32] Kew Y., Tan C. Y., Ng C. J. (2017). Prevalence and associations of neuropathic pain in a cohort of multi-ethnic Asian low back pain patients. *Rheumatology International*.

[33] Duarte R. V., Raphael J. H., Dimitroulas T. (2014). Osteoarthritis pain has a significant neuropathic component: an exploratory in vivo patient model. *Rheumatology International*.

[34] Sofat N., Ejindu V., Kiely P. (2011). What makes osteoarthritis painful? The evidence for local and central pain processing. *Rheumatology*.

[35] Sonohata M., Tsuruta T., Mine H. (2014). Clinical characteristics of neuropathic pain in patients with carpal tunnel syndrome. *Hand Surgery*.

[36] Bouhassira D., Lantéri-Minet M., Attal N., Laurent B., Touboul C. (2008). Prevalence of chronic pain with neuropathic characteristics in the general population. *Pain*.

[37] Harifi G., Amine M., Ait Ouazar M. (2013). Prevalence of chronic pain with neuropathic characteristics in the Moroccan general population: a national survey. *Pain Medicine*.

[38] Ferini-Strambi L. (2017). Neuropathic pain and sleep: a review. *Pain and Therapy*.

[39] Attal N., Lanteri-Minet M., Laurent B., Fermanian J., Bouhassira D. (2011). The specific disease burden of neuropathic pain: results of a French nationwide survey. *Pain*.

[40] Cohen S. P., Mao J. (2014). Neuropathic pain: mechanisms and their clinical implications. *BMJ (Clinical research ed.)*.

[41] Arimura T., Hosoi M., Tsukiyama Y. (2012). Pain questionnaire development focusing on cross-cultural equivalence to the original questionnaire: the Japanese version of the Short-Form McGill Pain Questionnaire. *Pain Medicine*.

[42] Argoff C. E. (2007). The coexistence of neuropathic pain, sleep, and psychiatric disorders: a novel treatment approach. *The Clinical Journal of Pain*.

